# The palate and choanae structure of the *Susisuchus anatoceps* (Crocodyliformes, Eusuchia): phylogenetic implications

**DOI:** 10.7717/peerj.5372

**Published:** 2018-08-10

**Authors:** Karla J. Leite, Daniel C. Fortier

**Affiliations:** 1 Departamento de Geologia, Universidade Federal do Ceará, Fortaleza, Ceará, Brazil; 2 Departamento de Ciências Biológicas, Universidade Federal do Piauí, Floriano, Piauí, Brazil

**Keywords:** Crocodyliformes, Eusuchia, Lower Cretaceous, Araripe Basin

## Abstract

Crocodyliformes is a group with a broad fossil record, in which several morphological changes have been documented. Among known transformations the most iconic is perhaps the series of changes seen in the structural evolution of the choanae. The change in the position of the choanae was important during the evolutionary history of the Crocodyliformes. This structure is relevant in the phylogenetic position of many crocodyliforms. The new skull of *Susisuchus anatoceps* from the Crato Formation of the Santana Group (Lower Cretaceous) is described and the preservation in the ventral view allows character encoding not yet observed for the species. The new specimen shows a typical eusuchian palate for *Susisuchus anatoceps*, in which the choana is fully enclosed by the pterygoid. The Susisuchidae clade has been placed in different phylogenetic positions: as a sister group of Eusuchia, advanced Neosuchia and in Eusuchia. In *Isisfordia* there are reports that the choana of this taxon is or is not fully enclosed by the pterygoid. The encoding of the ventral characters of *S*. *anatoceps* places Susisuchidae in Eusuchia. However, this position must be further studied, since the matrices showed fragility in the reconstitution of the Neosuchia–Eusuchia transition.

## Introduction

The crocodyliforms passed through some morphological changes. One of the most important modification examples is the palate and choanae structures during their evolutionary history (e.g., Notosuchia, there are occurrences of different patterns of subrectangular, elliptical, long and narrow choana; Andrade, Bertini & Pinheiro, 2006). The progressive change in choana position is related to the position of different palatal bones (Pol, Turner & Norell, 2009), probably due to the need of decoupling the oral cavity from respiration (Turner & Buckley, 2008).

In Crocodyliformes, the anterior margin of the choana has been presented in three different ways: limited by the maxilla (for example, *Goniopholis*), formed by palatines (for example, *Sarcosuchus imperator* Broin & Taquet, 1966) and anterior margin formed by pterygoids (e.g., *Hylaeochampsa vectiana* Owen, 1874) (Sereno et al., 2001; Pol, Turner & Norell, 2009). The location of the choanal opening varies from an anterior position in basal forms to a posterior position in modern crocodyliforms (Huxley, 1875; Colbert, 1969; Langston, 1973; Buffetaut, 1979; Clark, 1994; Brochu, 2003).

Choana position is one of the fundamental characters of phylogenetic positioning of many groups. In Eusuchia, the procoelous vertebrae and choana fully enclosed by the pterygoid bone are traditional morphological characteristics (Huxley, 1875; Benton & Clark, 1988; Clark, 1994).

The Susisuchidae clade has been placed in different phylogenetic positions and has been considered as a sister group of Eusuchia (Fortier & Schultz, 2009) inserted within the eusuchians (Andrade et al., 2011) and among advanced neosuchians (Turner & Pritchard, 2015). This clade is part of an important evolutionary context, possibly when groups of advanced neosuchians developed a complete secondary palate, with the choana entirely positioned in the pterygoid.

The Susisuchidae position is sensitive to the interpretation of the structure of the choana, with alterations of the topologies of the trees due to change of few characters. For example, the choana of *Isisfordia* (Turner & Pritchard, 2015) demonstrate the instability in Neosuchia relations. The new Cretaceous susisuchids of the Araripe Basin described here show the position of the choana between the early Neosuchia condition and the classic Eusuchia condition.

In this work, we will describe a new skull of *Susisuchus anatoceps*, in ventral view, allowing the encoding of the palatal characters for this taxon. These characters were not encoded for the holotype. A revised phylogenetic analysis incorporating this new morphological information will give a more comprehensive understanding of the phylogenetic positioning for Susisuchidae.

## Geological Setting

The Araripe Basin is included in a set of small inland basins of the northeast of Brazil, located in the extreme south of the State of Ceará, also comprising portions of the states of Pernambuco and Piauí. The origin and evolution of the Araripe Basin are related to the Gondwana fragmentation and the opening of the South Atlantic Ocean (Valença, Neumann & Mabesoone, 2003). Analysis with dating aid and using microfossils point to a polycyclic sedimentary history, comparable to some intracratonic basins and continental margin basins (Arai, 2006).

**Figure 1:**
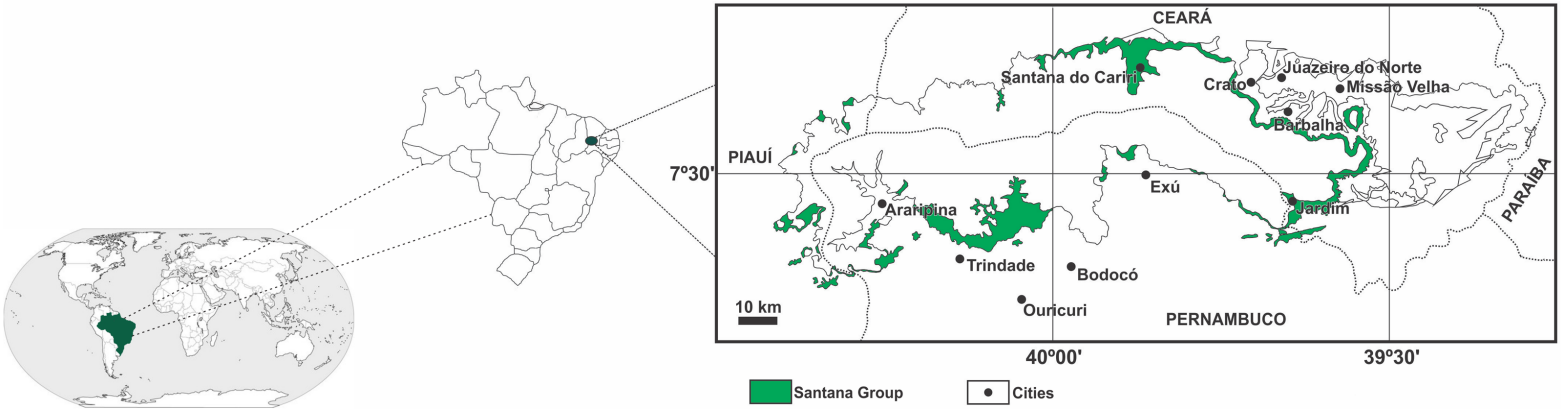
Location of the Araripe Basin. In featured (green) the Santana Group. Modified from Assine (2007).

The sedimentary sequence of the Araripe Basin dates from the Paleozoic and Mesozoic periods. The most representative Mesozoic sequence is the Santana Group (Fig. 1), which shows the record of the implantation of the first lacustrine system in the basin. This is characterized by anoxia conditions which favored the preservation of a large amount of organic matter (Assine, 2007).

The Santana Group is constituted from the base to the top by the Barbalha, Crato, Ipubi and Romualdo formations (Neumann, 1999; Assine et al., 2014). The group is known worldwide for the diversity and quality of preservation of its fossiliferous content, mainly from the Crato Formation.

The Crato Formation consists predominantly of light gray and cream-colored laminate limestones with intercalations of laminated carbonate clay and bituminous pelitic levels deposited in a lacustrine environment (Assine, 2007).

Preserved biota in the Crato Formation comprises representatives of invertebrates, vertebrates, plants and microfossils. The vertebrates are represented by fish, anurans, turtles, lizards, pterosaurs, birds and crocodilians (Brito, 2005; Moura & Báez, 2006; Oliveira, 2007; Frey & Salisbury, 2007; Figueiredo et al., 2011; Carvalho et al., 2015). Some specimens of *Susisuchus anatoceps* and one of *Araripesuchus* sp. were reported for the Crato Formation (Salisbury et al., 2003; Frey & Salisbury, 2007; Figueiredo & Kellner, 2009; Figueiredo et al., 2011; Field & Martill, 2017).

The *Susisuchus anatoceps* species is inserted in an important evolutionary context, when the advanced neosuchians groups developed a complete secondary palate, with the choana entirely positioned in the pterygoid. This specimen described here, preserved in the ventral view, has characters not encoded for the holotype and brings a more comprehensive understanding of the phylogenetic positioning for the Susisuchidae clade.

## Material and Methods

The material described here belongs to the collection of the Fundação Paleontológica Phoenix, under number FPH-243-V. The material was collected in calcareous quarries in the municipality of Nova Olinda, in the Northeast of Brazil.

The specimen is almost complete, with the skull and many postcranium elements. In this paper, the skull and the first cervical vertebrae are described. Until now, it has not been possible to prepare the postcranium elements.

The fossil lies on a plate of cream-colored limestone, with many white filaments, probably algal remains. To remove the sediment and better expose the preserved bones, a mechanical preparation of the fossil was performed using needles and dental equipment.

The skull has an elongated shape: 12.5 cm in length from premaxilla to atlas and 4.5 cm maximum width. Most bones are preserved. Premaxilla, maxilla, palatine, suborbital fenestra, pterygoid, quadrate, ectopterygoid, basisphenoid, basioccipital, hyoid, teeth and mandible are recognisable in the ventral view. The first cervical vertebra can also be identified.

## Systematic Palaeontology

**Table:** 

CROCODYLOMORPHA Walker, 1968
CROCODYLIFORMES Benton & Clark, 1988
MESOEUCROCODYLIA Whetstone and Whybrow, 1983
EUSUCHIA Huxley, 1875
SUSISUCHIDAE Salisbury et al., 2003
*Susisuchus anatoceps* Salisbury et al., 2003

**Diagnosis**: *Susisuchus anatoceps* is distinguished from all other neosuchians by containing the following combination of osteological features: posterior process of the maxillary bone separating lacrimal from nasal; lacrimal extends anteriorly beyond the anterior limit of the prefrontal; needle-like and homodont teeth; lateral margins of the frontal are elevated, forming ridged orbital margins; scapular blade has straight anterior and concave posterior margins; 10 or 11 thoracic vertebrae; four lumbar vertebrae; minimum width of the sacral ribs in the anteroposterior direction exceeds the maximum width of any of the transverse processes; postzygapophyses of caudal vertebrae VI–XI (the vertebrae terminal to caudal XI are not preserved) unite medially to form a flat, horizontally aligned shelf, which extends terminally over the vertebral foramen; maximum width of the proximal extremity of the ulna equivalent to that of the distal extremity, and slightly less than twice the minimum thickness of the ulnar shaft; absence of an anterior tubercle on the proximal extremity of the ulna; unguals present only in hand digits I and II; dorsal shield comprising two rows of paravertebral osteoderms and two left and two right rows of accessory osteoderms and amphicoelous thoracic, lumbar, and caudal vertebrae.

**Figure 2:**
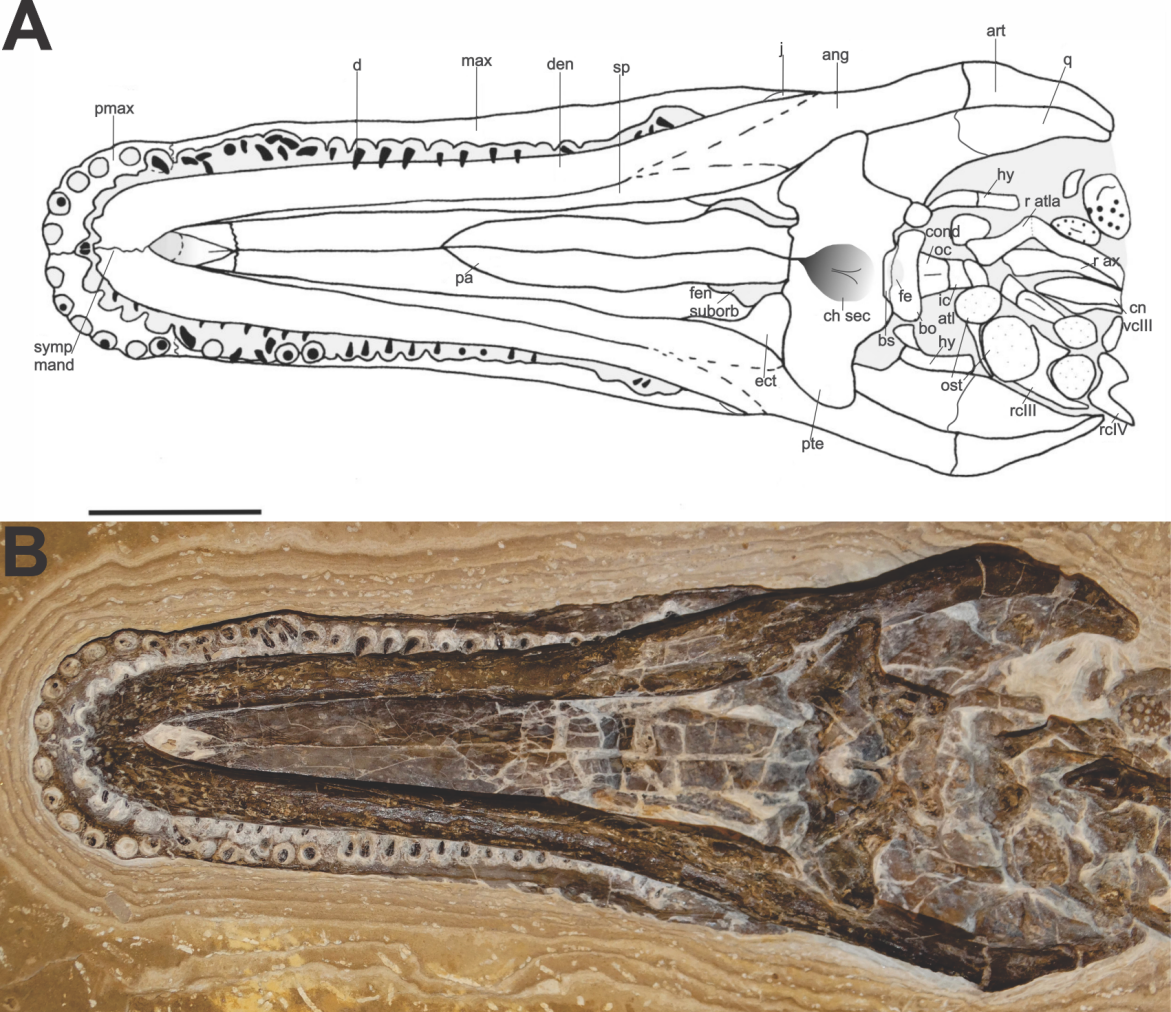
Specimen skull *Susisuchus anatoceps* (FPH-243-V) ventral view exhibit the secondary palate. Schematic diagram (A) and photograph (B). Abbreviations: ang, angular; art, articular; bo, basioccipital; bs, basisphenoid; ch sec, secondary choanae; cn vcIII, centrum cervical vertebrae III; cond oc, occipital condyle; d, dentary; den, tooth; ect, ectopterygoid; fen suborb, suborbital fenestra; fe, eustachian foramen; hy, hyoid; ic atl, atlas intercentrum; j, jugal; max, maxila; ost, osteoderm; pa, palatine; pmax, premaxilla; pte, pterygoid; q, quadrate; r atl, ribs atlas; r ax, ribs axis; r cIII, ribs cervical III; r cIV, ribs cervical IV; sp, splenial; symp mand, mandibular symphysis. Scale 2 cm. Photograph and schematic diagram credit Karla J. Leite.

**Description.** The comparison of the specimen to the two reported crocodilian genera of the Crato Formation (Salisbury et al., 2003; Frey & Salisbury, 2007) suggests that FPH-243-V is a *Susisuchus anatoceps*. The holotype of *Susisuchus anatoceps* is preserved in dorsal view, so its diagnosis is based on the observation of the bones dorsally. Thus, it is difficult to compare the description of the holotype and the ventrally preserved bones in the example described herein (Fig. 2). A character similar to the diagnosis of holotype is the shape of the teeth. Both have homodont, needle-shaped teeth. The specimen has a long maxillary rostrum and relatively flat lateral margin, with a platyrostral shape, as observed in the other Susisuchidae specimens. In this work we will consider the diagnosis of *S. anatoceps* characters of the holotype diagnosis (Salisbury et al., 2003) plus the characters added in the description of the MPSC-R1136 (Figueiredo et al., 2011).

Skull size and bone ossification suggest it is an adult *Susisuchus*. This contradicts the work of Sayão et al. (2016), who considered the adult individual of this species as relatively small. Previous studies have shown smaller size specimens as juvenile forms, individuals not yet completely mature (Salisbury et al., 2003; Figueiredo et al., 2011).

**Skull.** The premaxilla is partially covered by the dentary, with only a small palatal part and dental alveoli visible. The premaxilla-maxilla suture is a butt joint, as in many Crocodyliformes. This suture has a sinusoidal orientation in palatal view, as in the eusuchians *Alligator* and *Shamosuchus*, and differs in *Isisfordia*, which has the straight premaxilla-maxilla suture in palatal view. The ventral edge of premaxilla is located at the same height as the ventral edge of maxilla, common in many Crocodyliformes. The anterior alveolar margin of premaxilla has vertical orientation.

Most premaxillary teeth were lost, preserving only a single tooth and a few fragments present within multiple alveoli. The tooth row of the premaxilla is posterolaterally oriented. It is noticeable that the teeth present similar sizes, without any procumbency and ventrally orientated. The last premaxilla tooth is small and pointed, similar to the first maxillary tooth, as in *Isisfordia*, *Acynodon iberoccitanus* (Buscalioni, Ortega & Vasse, 1997) and *Pachycheilosuchus trinquei* (Rogers, 2003) (see Rogers, 2003; Martin, 2007).

On the palate, the foramen located on premaxilla-maxilla suture near the alveolar border is absent. This absence is common in many eusuchians, such as *A. iberoccitanus*. Although all teeth are not preserved, it is noticeable that the specimen has six premaxillary teeth, since the six dental alveoli are well visible. This same amount of premaxillary teeth is seen in the advanced neosuchians *Oceanosuchus boecensis* (Hua et al., 2007), *Meridiosaurus vallisparadisi* (Mones, 1980) and *Elosuchus cherifiensis* (Lavocat, 1955) (see Fortier, Perea & Schultz, 2011; Martin & Buffetaut, 2012). This characteristic differs from the description of the holotype of *Susisuchus anatoceps* and the *Isisfordia*, which presents five teeth (Salisbury et al., 2003; Salisbury et al., 2006). The number of premaxillary teeth may be an intraspecific variation in susisuchids.

There is a part of the incisive foramen visible in the posterior region of the premaxilla, almost in the suture between premaxilla and maxilla. The foramen narrows posteriorly in the posteromedial direction of the premaxilla. The posterior part of the incisive foramen is located medially in the premaxilla, possibly between the first dental alveoli. This position is not precise since the anterior part of the foramen is under the dentary.

The maxilla is long and presents a relatively flat margin with some small depressions of the lateral surface. On both sides the maxilla is preserved, and the ventral region of the dental alveoli and the palatal region are visible. The ventral edge of maxilla is straight in lateral view. The maxillary teeth are homodont and needle shaped, as observed in the holotype of *Susisuchus anatoceps* (Salisbury et al., 2003).

The teeth are still preserved in isolated dental alveoli. There are about 20 teeth on each side of the maxilla. The maxillary teeth are equal in size to the premaxillary teeth. There is only a single cusp on the teeth and its enamel surface is smooth.

Posterolateral to the maxilla, a small contact with the jugal is visible. However, most of the jugal is inside the limestone, and detailed description of this bone is not possible.

The ectopterygoid is preserved in articulation with the pterygoid, but much of it is covered. The contact between the ectopterygoid and pterygoid, in ventral view, occupies more than half of the expansion of the medial lateral border of the pterygoid. Although hardly visible, the ectopterygoid forms the lateral border of the suborbital fenestra. The ectopterygoid there has no participation in the palatine bar, just as it does not in the eusuchians *Acynodon adriaticus* (Delfino, Martin & Buffetaut, 2008). Like most Crocodyliformes the ectopterygoid does not extend to the posterior tip of the lateral pterygoid.

The quadrate is relatively broad and has a significant ventral process on lateral braincase wall. The major axis of the quadrate is directed posteroventrally, and the condyles are closely aligned horizontally. These features are observed in *Susisuchus jaguaribensis* (Fortier & Schultz, 2009) and are common to Crocodyliformes, but differ from those found in notosuchians, like *Uruguaysuchus aznarezi* (Rusconi, 1933); *Baurusuchus pachecoi* (Price, 1945) and *Sphagesaurus huenei* (Price, 1950) which has the major axis of the quadrate ventrally directed (see Pol, 2003; Pol & Apesteguía, 2005). The pterygoid process of the quadrate is well developed, with some striations, and is in contact with the pterygoid. There is a crest that divides the ventral margin of the quadrate into two convex parts, a part oriented anteriorly to the dorsal region and a deeper posterior part. The concavity of the crest disappears progressively posterolaterally.

The mandibular condyle of the quadrate is in contact with the articular bone. The articular facet for quadrate condyle is equal in length to the quadrate condyles. This character differs in *Isisfordia*, where the articular facet for quadrate condyle is slightly longer. The quadrate mandibular condyles are in position beyond the level of the occipital condyle. The jaw joint, inferred by the position of the quadrate articular condyles, is positioned at the level of the basioccipital condyle. This condition is found in advanced neosuchians and eusuchians.

The palatine presents a flat surface, maintaining a constant width throughout the length, without expansion in the region of contact with the pterygoid as it occurs in *Isisfordia* and other advanced neosuchians (Pol, Turner & Norell, 2009). The medial suture is well marked on the anterior extremity, where there is contact with the maxilla through a V-shaped suture. This character is similar in *Acynodon* and differs in *Isisfordia*, whose maxilla-palatine suture has an anteriorly rounded shape. Posteriorly the palatine is firmly connected to the pterygoid by a transverse suture, like most Crocodyliformes, except some more basal like the protosuchians e.g., *Edentosuchus tienshanensis* (Young, 1973) and *Protosuchus richardsoni* (Brown, 1933), in which the palatines overlie the pterygoids (Pol et al., 2004). Posterolaterally, the palatine constitutes the border of the suborbital fenestra. The suborbital fenestrae are preserved, they just appear covered up by the dentaries.

The pterygoid is broad and has a smooth palatal surface. It is articulated with both the quadrate and basisphenoid. The pterygoidean flanges are thin, laminar and expanded, as in *Isisfordia*. In ventral view it has participation in the suborbital fenestra, as in most crocodyliforms (Brochu, 1999). The pterygoid ramus of the quadrate has a flat ventral edge. This character is observed in many crocodyliforms, with some exceptions like the eusuchians *Borealosuchus formidabilis* (Erickson, 1976) and *Leidyosuchus canadensis* (Lambe, 1907), which have pterygoid ramus of quadrate with deep groove along ventral edge (Brochu, 1999).

The pterygoid is mediolaterally broad, reaching laterally beyond the medial margin of quadrate condyles, similar to many Crocodyliformes. The character is different in some eusuchians e.g., *Acynodon* sp. and *H. vectiana* in which the relatively narrow pterygoid flange shape does not reach laterally to medial margin of quadrate condyles.

The pterygoids are fused posterior to the choana, like many crocodyliforms, except for some basal forms that have the pterygoids unfused posteriorly, as *Terrestrisuchus gracilis* (Crush, 1984) and *Orthosuchus stormbergi* Nash, 1975 (Pritchard et al., 2013; Irmis, Nesbitt & Sues, 2013).

**Figure 3:**
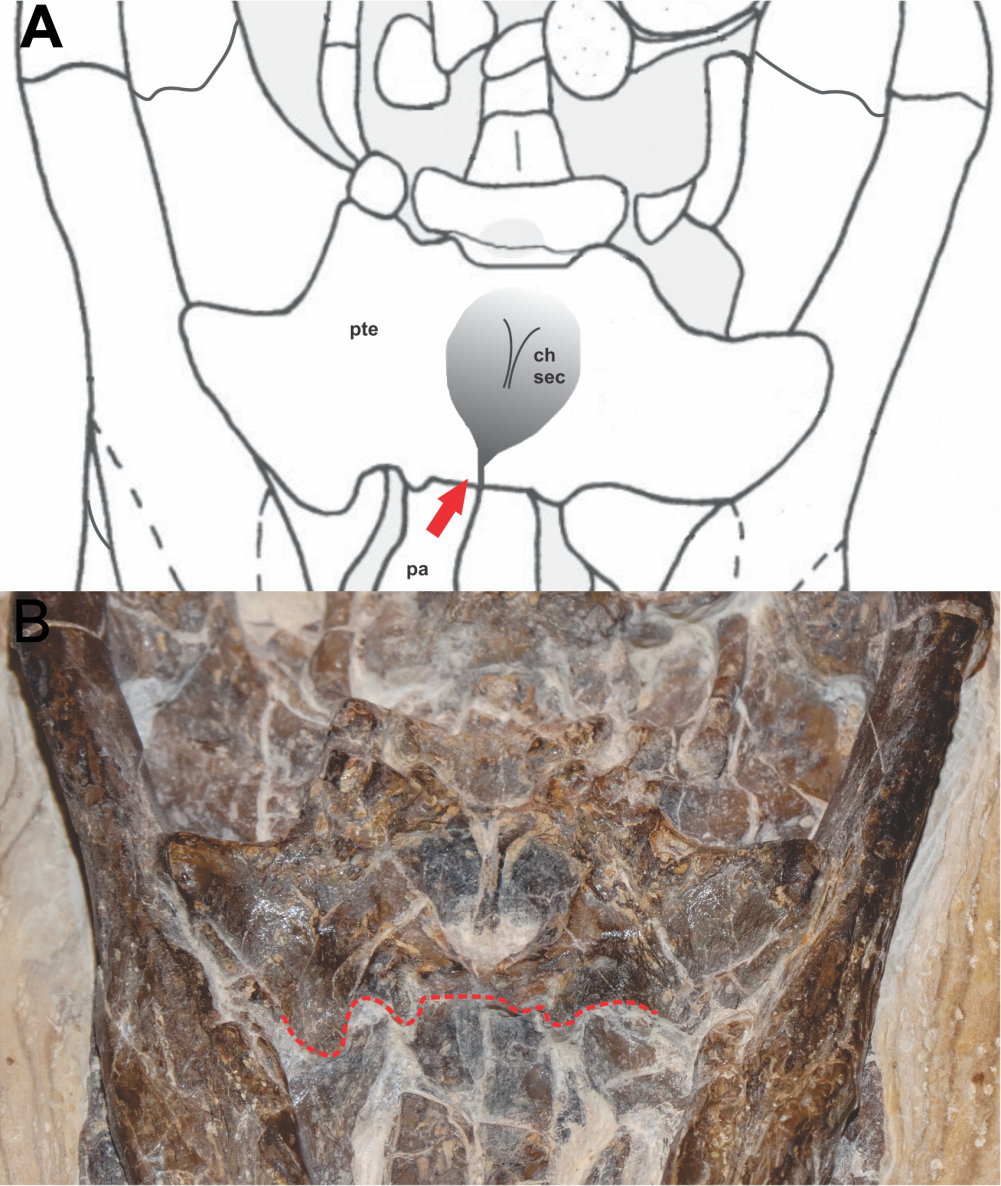
Detail of the position of the choana in FPH-243-V. (A) Schematic diagram and (B) photograph. Line and arrow in red indicates the anterior border of the pterygoid, in the palatine-pterygoid suture. Photograph and schematic diagram credit Karla J. Leite.

The choana is well preserved, completely encased in the primary pterygoidean palate (Fig. 3). Such a characteristic is common in Eusuchia as in *A. adriaticus, A. iberoccitanus, H.  vectiana* and *Iharkutosuchus makadii* (Ösi, Clark & Weishampel, 2007; Brochu, 1999). There is no depression posterior to the choana on the primary pterygoid palate, as in almost all Crocodyliformes, except some notosuchians, for example, *B. pachecoi* and *Comahuesuchus brachybuccalis* (Bonaparte, 1991; Pol et al., 2014). The choanal opening opens into the palate through a deep midline depression (choanal groove), as observed in most Crocodyliforms, with the exception of the most basal, such as *Protosuchus* and *Hemiprotosuchus*. The choana is posteriorly closed by an elevated wall formed by pterygoids, like most crocodyliforms, excluding basal forms and the eusuchian *Argochampsa krebsi* (Hua & Jouve, 2004).

The anterior edge of choana is situated near the posterior edge of the suborbital fenestra. This character is common in many eusuchians, as in Glen Rose form (Langston, 1973); *Bernissartia fagesii* (Dollo, 1883); *Theriosuchus pusillus* (Owen, 1879); *Shamosuchus djadochtaensis* (Mook, 1924) and *Shamosuchus major* (Efimov, 1981) (Pol, Turner & Norell, 2009; Turner, 2015). However, it differs in *Isisfordia*, which has the anterior edge of choana placed between the suborbital fenestra (Turner & Pritchard, 2015). The posterior margin of the choanal groove is posteriorly located on the pterygoids near the posterior margin of these bones, common character in neosuchians and eusuchians.

The choana has a mediolateral width similar to the minimum mediolateral width of the palatine, which differs from *Isisfordia* and other neosuchians in the maximum mediolateral width of the palatine (Salisbury et al., 2006). The choanal groove is completely septated by a narrow-shaped septum vertical bony sheet. This septum is smooth, and narrow vertical bony sheet, as seen in *Isisfordia* and *B. fagesii*. This complete separation of the choana also occurs in *Isisfordia*, advanced neosuchians (e.g., *Goniopholis simus* Owen, 1878 and *Sunosuchus junggarensis* Wu, Brinkman & Russell, 1996) and eusuchians (e.g., *S. djadochtaensis* and *Alligator mississippiensis* Daudin, 1802) (Pol, Turner & Norell, 2009; Pritchard et al., 2013; Turner, 2015). The palatine-pterygoid contact is anteriorly positioned to the choana. This character varies among the eusuchians. Some are similar, such as *A. iberoccitanus* and *Allodaposuchus precedens* Nopcsa, 1928 (Martin, 2007; Martin, 2010). In other eusuchians the pterygoid-palatine contact is prong and the pterygoids extend into the bar between the suborbital fenestrae for example, in *I. makadii* and *H. vectiana* (Clark & Norell, 1992; Ösi & Weishampel, 2009)*.* The basisphenoid is medially positioned in the pterygoids, whose anterior border in contact with the pterygoid is convex. Ventrally, in the deep posterior region, it articulates with the basioccipital.

The basisphenoid is exposed on ventral surface of braincase without lateral exposure, similar to some eusuchians, such as *Theriosuchus guimarotae* (Schwarz & Salisbury, 2005) and *S. djadochtaensis* (Turner, 2015). This character differs in *Isisfordia* and in the neosuchians, in which the basisphenoid is virtually excluded from the ventral surface by pterygoid and basioccipital. The basisphenoid extends caudoventrally as a thin lamina between the basioccipital and pterygoid, with the medial eustachian opening lying between the basioccipital and the descending lamina of the basisphenoid. This character appears as the condition seen in *Hylaeochampsa* and in all mature crocodylians, except *Gavialis* (Brochu, 2004). The basisphenoid exposure is short and narrow, as the whole bone is much larger.

It is possible to observe a small part of the occipital condyle. Narrow tuberosities extend ventrolaterally from the occipital condyle. The medial foramen of the eustachian is relatively large and oval. The medial opening of the eustachian foramen lies ventrally in the basioccipital-basisphenoid suture. The depth of the median eustachian aperture varies in ontogenetic form. The anterior and posterior branches are externally visible in all hatchling crocodilians. In living crocodilians, the separation between branches is narrower in early ontogeny (Brochu, 1999). The median eustachian foramen is relatively large and with oval shape, with an anteroposteriorly directed axis. The aperture observed in the FPH-243-V is similar to the mature *A. mississippiensis* (Brochu, 1999). This comparison suggests that this new specimen is possibly an adult individual. However, only more detailed studies can identify the age of this individual.

The robust mandible occludes with the maxila. A mandibular fenestra is absent. This absence is common in most neosuchians related to Eusuchia (e.g., *S. djadochtaensis*, Glen Rose form, *B. fagesii*, *T. pusillus* and *Rugosuchus nonganensis* (Wu, Cheng & Russell, 2001) (see Pol, Turner & Norell, 2009). The posterior region of the mandible is slightly wider. The well preserved mandibular symphysis is visible in the anteromedial region of the dentary. The mandibular symphysis is relatively sigmoid, narrow and shallow. It extends to the fourth tooth. The posteroventral edge of mandibular ramus has a convex shape, like most Crocodyliformes.

The dentary has a relatively ornate surface. This ornamentation forms a bumpy pattern with small depressions, as in the holotype of *Susisuchus anatoceps* (Salisbury et al., 2003). The ventrolateral margin has a convex shape, as in most neosuchians (Pol, Turner & Norell, 2009; Ortega et al., 2000). The dentary articulates medially with the splenial and posteriorly with the angular.

Anteriorly, the height of the dentary is relatively low, forming a short shallow mandibular symphysis, as in other neosuchians (e.g., *Shamosuchus*) (Pol, Turner & Norell, 2009; Turner, 2015). In ventral view the dentary symphysis has lateral edges longitudinallly oriented, convex anterolateral corner, and extensive transversally oriented anterior edge, similar to *Simosuchus clarki* (Buckley et al., 2000).

The splenial-dentary suture is linear, V-shaped, along the mandibular branch, extending along the edge of the teeth. This transverse suture is similar to the notosuchians, like *Sphagesaurus montealtensis* (Andrade & Bertini, 2008) and *Yacarerani boliviensis* (Novas et al., 2009). The mandibular teeth are apparently homodontes and they are barely visible.

The splenial narrows anteriorly, tapering posterior to the mandibular symphysis. The splenials end before the anterior extremity of the mandible, at the point of contact between the premaxilla and the maxilla. The splenials do not participate in the mandibular symphysis, as observed in *Isisfordia* (Salisbury et al., 2006). The entire medial surface of the splenials is smooth, and the posterior region is in contact with the anteromedial surface of the angular. The surface of splenials posterior to symphysis is flat and thin.

The angular is exposed in the ventral view, with a slightly arched anteroposterior contour. This bone is relatively long, with a convex medial contour. Anteriorly, the angular articulates with the dentary and the splenial in a V-shaped contact and posteriorly there is a small articulation between angular and articular. In the posterior region of the mandible there is a small part of the surangular exposed in the medial region under the angular. The retroarticular process is posteriorly elongated in triangular-shaped and dorsally facing the skull.

The surangular is smooth, with a small region of contact with the articular. The articular is relatively short and medially curved, with a transverse ridge that separates the posterior region from the anterior. The articular is more expanded anteriorly in the region, where it lies above the posterior margin of the quadrate. The anterior extremity of the articular is rod-shaped, as in *S. djadochtaensis* and most crocodyliforms (Pol, Turner & Norell, 2009). The articular lacks medial process as in many Crocodyliformes.

**Hyoid**. The ceratobranchial of the hyoid apparatus is well preserved. This bone is thin with slightly expanded ends. The ceratobranchial is slightly curved anteromedially. In ventral view, the ceratobranchial extends from the posterior border of the pterygoid to the end of the cranial cavity. Records of preserved hyoid in fossil crocodilians are rare.

**Cervical vertebrae.** It is possible to identify the first cervical vertebrae atlas-axis still articulated to the skull. The ribs of the atlas, axis and the cervical ribs III and IV are visible. The atlantal intercentrum is in contact with the occipital condyle. Also, part of neural centrum of the CIII is visible. Near the ribs some ventral osteoderms are present.

The ribs of the atlas have only one contact surface at the proximal end. This surface fits into the intercentrum of the atlas. The ribs are sword-shaped, long and narrow transversely. The ribs of the axis also have the shape of a sword blade, being similar in shape to the rib of the atlas, except for the proximal end, which possesses a short tuberculum and a capitulum, which are still articulated with the vertebra. This structure is intermediate between the pair of atlas ribs and the other cervical ribs. In these, both the capitular and the tubercular articulate with their respective vertebrae. The rib of the shaft is slightly shorter than the rib of the atlas.

Cervical ribs III and IV are very similar. Both have the same shape, and the IV cervical rib is more robust. Each rib consists of an axis that extends horizontally, parallel to the vertebral column, and another axis with the tuberculum and the capitulum articulated to the vertebrae. The body of rib III overlaps the anterior projection of rib IV. Between these ribs there is a small ventral osteoderm.

## Position of Choana and Morphology of Vertebrae

Choana position in Crocodyliformes has gradually changed in the evolutionary history of the group. The position of the choana shifted from an anterior location in basal forms to a posterior placement in modern crocodyliforms (Eusuchia). Considering this gradual change in choana position, there was also a change in palatine bones. Pol, Turner & Norell (2009) consider three evolutionary degrees: the protosuchian condition (anterior margin limited by the maxilla), the mesosuchian condition (anterior margin formed by palatine) and the eusuchian condition (anterior margin formed by the pterygoids). According to this classification, based on the specimen described herein, the position of the choana in *Susisuchus anatoceps* is classified as an eusuchian condition.

The specimen FPH-243-V has the palatal region quite visible and the position of the choana is very perceptible, completely enclosed by the pterygoid, with the anterior borders of the choana formed by the pterygoids and located after a palatine-pterygoid transversal suture.

The susisuchids *Isisfordia* and *Susisuchus* seem to differ in choana position. Based on the holotype (QM F36211) and paratype (QM F44320, QM F44319 and QM F34642; Salisbury et al., 2006) reconstituted *Isisfordia* with the contact suture of the anterior palatine-pterygoid of the choana. The involvement of pterygoids in the secondary bony palate of *Isisfordia* is minimal when compared to extant Crocodylia, including *Gavialis*. These authors consider *Isisfordia duncani* as a sister taxon of *Hylaeochampsa* and Crocodylia, and these sister groups of *Susisuchus*. The synapomorphies that unite *Isisfordia* to *Hylaeochampsa* and Crocodylia are secondary choanae closed by the ventral lamina of the pterygoid and the cervical, thoracic and lumbar procoelous vertebrae. The combination of these characters was pointed out with characteristics of the eusuchians (Benton & Clark, 1988; Brochu, 1999). Therefore, *Isisfordia* is placed with a basal taxon of Eusuchia (Salisbury et al., 2006).

Turner & Pritchard (2015) examined the holotype and all paratypes of *Isisfordia duncani* and considered that the choana of this taxon is not fully enclosed by pterygoid. For these authors, the palatal contributes considerably to the trailing edge of nasopharyngeal passage and laterally in contact with the pterygoid, still forming the anterior margin gently curved of the choana, showing an intermediate neosuchian condition. These authors, in relation to the vertebrae, observe a subtle posterior convexity in the center of some preserved cervical vertebrae. Considering the procoelous or “incipient procoely” vertebrae, as Figueiredo et al. (2011) observed in a specimen of *Susisuchus anatoceps*, the phylogenetic position of Susisuchidae does not change.

In *Susisuchus anatoceps* the position of the choana was unknown. However, the preservation in the ventral view of this new specimen (FPH-243-V) shows that the primary pterygoidean palate completely encloses the choana.

Therefore, the position of the choana in *Susisuchus anatoceps* is undoubtedly within the pterygoid, similar to eusuchians like *Allodaposuchus precedens*, *H. vectiana*, *I. makadii* and *Acynodon* sp. Clark & Norell, 1992; Buscalioni et al., 2001; Martin, 2007; Ösi & Weishampel, 2009; Martin, 2010.

However, the condition in *Susisuchus anatoceps* seems to differ substantially from the state in other eusuchians. In those taxa, the pterygoids meet at a relatively elongate, anteroposteriorly oriented suture. Nonetheless, *Susisuchus* seems to possess a small, anteroposteriorly short contact, as an intermediate stage between no contact and the broad contact in other Eusuchia.

Regarding the vertebrae, *Susisuchus* have the procoelous cervical vertebrae (or incipient procoely) with only the last platycoelous cervical vertebra and all amphioelous thoracic vertebrae (Salisbury et al., 2003; Figueiredo et al., 2011). These characters differ from Eusuchia whose cervical and thoracic vertebrae are procoelous (Brochu, 1999). The *Susisuchus* presents features demonstrating that the emergence of Eusuchia was not a simple event and is not fully understood.

## Phylogenetic Relationships

We performed a phylogenetic analysis using TNT v. 1.5 (Goloboff, Farri & Nixon, 2003). This analysis used the data matrix of Turner & Pritchard (2015) and included the FPH-243-V and the holotype characters coding of *Susisuchus anatoceps*. We used the Turner & Pritchard (2015) matrix because it is the most recent work that includes the clade Susisuchidae. The characters coding are listed in Supplemental Information 1 and 2. We performed a heuristic tree search strategy and performing 1,000 replicates followed by TBR branch swapping (holding 10 trees per replicate).

The first analysis considered the position of the choanae in the susisuchids. *Susisuchus* has an eusuchian-style palate (43.1) and *Isisfordia* has a non-eusuchian palate (43.0) (Turner & Pritchard, 2015). The matrix has a total of 109 taxa and 321 characters (some multistate and ordered). The objective of the first analysis was to test if the position of the choanae changes the relation between the species of susisuchids. In the matrix, *Susisuchus* and *Isisfordia* are incipient procoely (92.2). This analysis resulted in 108 most parsimonious trees of 1675 steps (CI = 0,24, RI = 0,68).

The coded characters for *Susisuchus anatoceps* were considered, in all the following analyses: an eusuchian type palate (43.1) as seen in FPH-243-V and procoelous cervical vertebrae (92.1) and amphicoelous trunk vertebrae (93.0).

**Figure 4:**
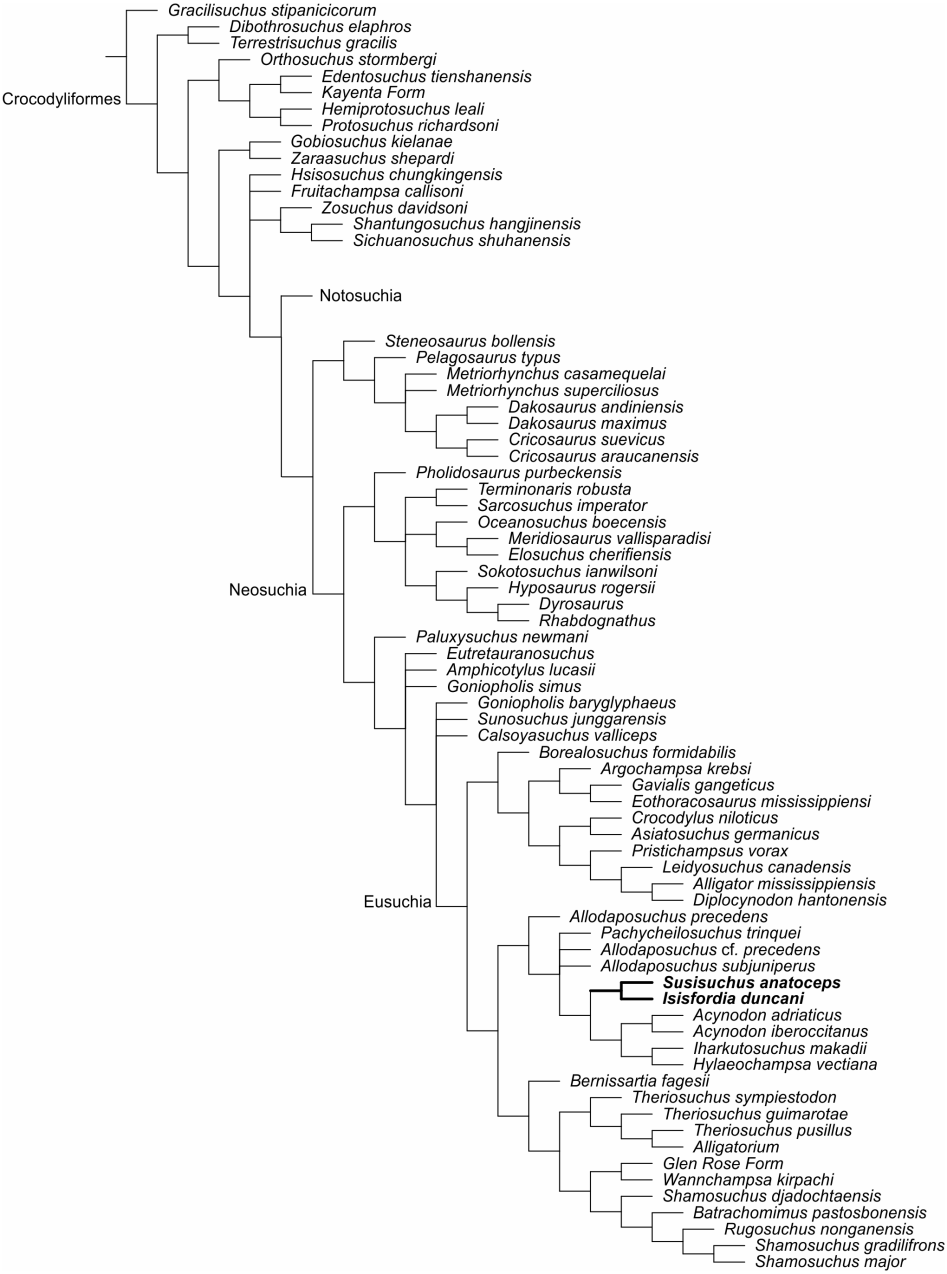
Strict consensus of the second analysis in the matrix of Turner & Pritchard (2015) adding FPH-243-V and encoded characters for the holotype. The new position of the susisuchids between Eusuchia (Bold).

A second analysis was produced to test if the traditional interpretation of vertebral morphology affects the results. Therefore, vertebrae were only scored as either amphicoelous or procoelous, without the “incipient procoely” state. *Isisfordia* scored for a non eusuchian-style palate (43.0) (Turner & Pritchard, 2015) and it also scored for fully-developed procoely (92.1 and 93.1). The analysis resulted in 108 most parsimonious trees of 1,674 steps (CI = 0,24, RI = 0,7). Scoring of *Isisfordia* as derived for the amphicoelous trunk vertebrae (92.0 and 93.0) resulted in 108 most parsimonious trees of 1,675 steps (CI = 0,24, RI = 0,7) (Fig. 4).

A third analysis was produced to evaluate the interpretation of palate morphology presented by Salisbury et al. (2006). *Isisfordia* was coded for the condition of the eusuchian palate (43.1) and procoely condition (92.1 and 93.1). This analysis resulted in 108, most parsimonious trees of 1,673 steps (CI = 0,24, RI = 0,7).

A fourth analysis considers the interpretation of palate condition presented by Salisbury et al. (2006) and vertebral morphology presented by Turner & Pritchard (2015). Both susisuchids are eusuchian palate type (43.1). *Isisfordia* was coded as amphicoelous. This analysis resulted in 108, most parsimonious trees of 1,674 steps (CI = 0,24, RI = 0,7).

The same results were obtained in all analyses. Phylogenetic results of the reviews place *Susisuchus anatoceps* and *Isisfordia duncani* in a monophyletic group, Susisuchidae, similar to the analysis of Turner & Pritchard (2015). Four characters unite *Isisfordia* and *Susisuchus*, the choanal groove is completely septated (69.2), the dorsal osteoderms without articular anterior process (96.0), the maxillary teeth lateral compression symmetrically developed (140.2) and presence of pear shaped external naris (309.1).

The strict consensus places Susisuchidae within Eusuchia (Fig. 4). The encoding of palatal characters in *Susisuchus anatoceps* is fundamental for the inclusion of susisuchids among eusuchians. Without the coding of the ventral characters of FPH-243-V and its procoely condition of the cervical vertebrae, the clade Susisuchidae is considered an advanced Neosuchia (Fig. 4) (Turner & Pritchard, 2015).

Some characters place the susisuchids among the most derived eusuchians (e.g., *T. guimarotae*, *Shamosuchus* sp.) such as the nasal contributes to narial border (13.0), the absence of the mandibular fenestra (75.1) and the postzygapophyses of axis well developed and curved laterally (153.0). Two characters support a relationship between Susisuchidae to *A. iberoccitanus* and *I. makadii*: the lateral contour of snout in dorsal view is straight (178.0); and the presence of a midline crest on basioccipital plate below occipital condyle (297.1).

The analysis of *Susisuchus anatoceps* (excluding *Isisfordia* from the matrix) places this taxon in Eusuchia. It is perceptible that *Susisuchus* has transition characteristics between advanced Neosuchia and Eusuchia. It presents the choana enclosed by the pterygoid observed in FPH-243-V. The MPSC-R1136 specimen shows some procoelous cervical vertebrae and the amphicoelous thoracic vertebrae, interpreted as an early stage of the transition to procoely condition (Figueiredo et al., 2011).

Although the palatine condition in *Susisuchus* is clearly intermediate in structure between neosuchians and “advanced” eusuchians, it is worth noting that Susisuchidae nests within a clade of derived neosuchians with pterygoid-enclosed choanae in their figured phylogenetic analysis.

## Conclusions

FPH-243-V is the first described skull of the *Susisuchus anatoceps* in ventral view. It clearly shows the position of the choana fully enclosed by the pterygoid. The numbers of teeth in the premaxilla is different from the holotype, which is probably an intraspecific variation. As already mentioned in other studies, phylogenetic analysis places *Susisuchus* and *Isifordia* in a monophyletic group Susisuchidae. Encoding new characters for *Susisuchus* places susisuchids within Eusuchia. Susisuchidae appears to be an anatomical intermediate between the early Neosuchia condition and the “advanced” Eusuchia condition. The phylogenetic position of Susisuchidae should be studied in more detail. The reconstruction of neosuchian phylogeny is currently unstable.

##  Supplemental Information

10.7717/peerj.5372/supp-1 Supplemental Information 1 New character coding (321 characters) of *Susisuchus anatoceps* based on FPH-243-V in addition to the holotype, following data from Turner & Pritchard (2015) Data used in the first analysis.

10.7717/peerj.5372/supp-2 Supplemental Information 2 New character coding (342 characters) of *Susisuchus anatoceps* based on FPH-243-V in addition to the holotype, following data from Turner & Pritchard (2015) Data used in analyses 2, 3 and 4.

## Institutional abbreviations

FPH: Fundação Paleontológica Phoenix
MPSC: Museu de Paleontologia de Santana do Cariri, Brazil
QM: Queensland Museum, Brisbane, Australia

## Additional Information and Declarations

### Data Availability

The following information was supplied regarding data availability: The material described here belongs to the collection of the Fundação Paleontológica Phoenix, Aracaju, Brazil (no FPH-243-V).
