# Supplementary material for: The palate and choanae structure of the Susisuchus anatoceps (Crocodyliformes, Eusuchia): phylogenetic implications
Source: PeerJ. 2018 Aug 10;6:e5372. doi: 10.7717/peerj.5372 (PMC6089207; doi:10.7717/peerj.5372)
Supplement: Supplemental Information 1 — Data used in the first analysis. [file peerj-06-5372-s001.pdf]

Supplement 1. New character coding (321 characters) of *Susisuchus anatoceps* based on FPH-243-V in addition to the holotype, following data from Turner & Pritchard (2015). Data used in the first analysis.

203?1 21101 1?00? 11000 10011 1?001 00010 02010 1011? 11??0 ???10 0???? ?1?0?
1312? 3?0?1 ?000? 01??? ???1? ?2002 02011 0??0? 000?1 0??00 ?1?10 00??1 111?0
????0 00012 011?0 ?0000 ???20 ??001 0?001 022?0 1?0?? ?100? 0?0?0 0?0?0 0?01?
?0010 00?11 10?00 0?0?0 00000 0000? 0?000 0001? 00000 0010? ?0?00 ?1000 00???
??0?1 1?01? 0?010 00??0 0?0?0 0?0?0 ?0000 0?1?? ??00? 00010 00011 1110? 0
